# Clearing an ESKAPE Pathogen in a Model Organism; A Polypyridyl Ruthenium(II) Complex Theranostic that Treats a Resistant Acinetobacter baumannii Infection in Galleria mellonella

**DOI:** 10.1002/chem.202203555

**Published:** 2023-01-12

**Authors:** Kirsty Smitten, Hannah M Southam, Simon Fairbanks, Arthur Graf, Adrien Chauvet, Jim A Thomas

**Affiliations:** ^1^ Department of Chemistry University of Sheffield Sheffield S3 7HF UK; ^2^ School of Bioscience University of Sheffield Sheffield S10 2TN UK

**Keywords:** antimicrobial, AMR, ESKAPE, ruthenium, theranostic

## Abstract

In previous studies we have described the therapeutic action of luminescent dinuclear ruthenium(II) complexes based on the tetrapyridylphenazine, tpphz, bridging ligand on pathogenic strains of *Escherichia coli* and *Enterococcus faecalis*. Herein, the antimicrobial activity of the complex against pernicious Gram‐negative ESKAPE pathogenic strains of *Acinetobacter baumannii* (AB12, AB16, AB184 and AB210) and *Pseudomonas aeruginosa* (PA2017, PA_ 007_ IMP and PA_ 004_ CRCN) are reported. Estimated minimum inhibitory concentrations and minimum bactericidal concentrations for the complexes revealed the complex shows potent activity against all *A. baumannii* strains, in both glucose defined minimal media and standard nutrient rich Mueller‐Hinton‐II. Although the activity was lower in *P. aureginosa*, a moderately high potency was observed and retained in carbapenem‐resistant strains. Optical microscopy showed that the compound is rapidly internalized by *A. baumannii*. As previous reports had revealed the complex exhibited no toxicity in *Galleria Mellonella* up to concentrations of 80 mg/kg, the ability to clear pathogenic infection within this model was explored. The pathogenic concentrations to the larvae for each bacterium were determined to be≥10^5^ for AB184 and≥10^3^ CFU/mL for PA2017. It was found a single dose of the compound totally cleared a pathogenic *A. baumannii* infection from all treated *G. mellonella* within 96 h. Uniquely, in these conditions thanks to the imaging properties of the complex the clearance of the bacteria within the hemolymph of *G. mellonella* could be directly visualized through both optical and transmission electron microscopy.

## Graphical abstract

A dinuclear polypyridyl Ru^II^ complex is highly active against the ESKAPE pathogens *Acinetobacter baumannii* and *Pseudomonas aeruginosa*. With a single dose it can be used to both directly monitor, and completely clear, infections caused by an AMR strain of *A baumannii* that proves to be highly virulent in the *Galleria mellonella* larva model when left untreated.

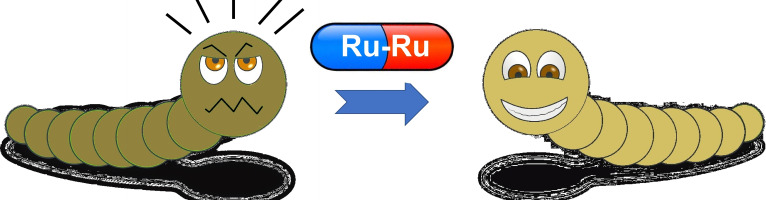

## Introduction

Antimicrobial resistance, AMR, is rapidly emerging as a healthcare and economic emergency, with only climate change presenting a bigger threat to global society.[^1^, ^2^, ^3^, ^4^] In fact, both climate change[^5^, ^6^, ^7^, ^8^] and the ongoing COVID19 pandemic,[^9^, ^10^, ^11^, ^12^] are exacerbating the emergence of therapeutically resistant pathogens. There is now a real threat that public health gains made over the last century in areas such as infant mortality may be reversed.^[13]^ In this context, many studies have highlighted particular concerns over the ESKAPE group of pathogens,[^14^, ^15^, ^16^] which produce the majority of nosocomial infections, and have been classified by the WHO as pathogens that urgently require the development of new treatments.[^17^, ^18^]

*Acinetobacter baumannii* ^[19]^ and *Pseudomonas aeruginosa* ^[20]^ are particularly problematic members of the ESKAPE group as many of their strains have intrinsic or acquired resistance to the majority of clinically available antimicrobial agents.[^21^, ^22^] While management of *A. baumannii* infections is becoming increasingly challenging due to its innate ability to survive in hospitals and persist on surfaces for extended periods of time,^[23]^ *P. aeruginosa* is a leading cause of nosocomial infections as it is responsible for 10 % of hospital‐acquired infections.^[20]^ Both bacteria cause a wide range of infections, including pneumonia, meningitis, urinary tract infections (UTIs), and bacteremia and sepsis that often result in threats to life.[^23^, ^24^, ^25^, ^26^] Although there is a critical need to develop and assess new treatments for these pathogens,^[27]^ and Gram‐negative bacteria in general,^[28]^ this goal has been hampered by several difficulties.

As the development of new molecular scaffolds will provide antimicrobials with new targets and activity, It is now widely acknowledged that the identification of novel therapeutics for Gram‐negative infections will require the investigation of wider chemical space.[^29^, ^30^, ^31^] Furthermore, several analyses have revealed that existing antibiotics active in Gram‐negative bacteria have quite distinctive chemical properties compared to typical therapeutics;[^32^, ^33^] for example, they tend to be more polar, more rigid, and less globular than Gram‐positive antibiotics, yet traditional medicinal chemistry has a bias toward less polar, more hydrophobic small molecules. Apart from the difficulty in designing new active molecules, the identification and development of promising antimicrobial leads through traditional in vitro screening methods is also problematic.

The activity of new leads can only be optimized if its therapeutic target is established, and its bacterial uptake is quantified. Consequently, a range of analytical methods to quantify the uptake of antibiotics have been reported. These methods either require specialized mass spectrometry techniques^[34]^ or the chemical derivatization of an already identified lead with a fluorophore.[^35^, ^36^, ^37^, ^38^]

A second common difficulty in screening arises from antimicrobial efficacy tests, such as disk diffusion assays, commonly used to assess in vitro therapeutic activity, as these methods quite often do not correlate with in vivo efficacies[^39^, ^40^] later assessed in animal models, such as mice and rats, which in themselves require costly and time‐consuming specialized facilities. In this context, *Galleria mellonella* (the Greater Wax Moth caterpillar) has recently emerged as a convenient and viable alternative for such studies.[^41^, ^42^] Its ethical and logistical advantages, as well as low costs, make *G. mellonella* an attractive model for the study of host‐pathogen interactions, especially as it has been widely established there is a striking correlation between bacteria virulence in mammals and *Galleria*.[^42^, ^43^] Indeed, *G. mellonella* has been used to study the dynamics and virulence of *P. aeruginosa*[^44^, ^45^] and *A. baumannii* infections.[^46^, ^47^]

Unlike other non‐vertebrate models – but similar to mammals – insects have a complex innate immune system comprised of humoral and cellular responses.^[48]^ As the larvae's hemolymph cells can phagocytose foreign microbial invaders and even produce antimicrobial peptides, this model provides antimicrobial defense information related to that observed in mammalian infection processes.[^49^, ^50^] Its easily accessible injection sites (pro‐legs) and incubation temperatures (37 °C) make *G. mellonella* a convenient infection model to study human pathogens.[^41^, ^42^, ^43^] So, it is unsurprising that *G. mellonella* is becoming increasingly employed in studies on the in vivo activity of novel antimicrobial agents, particularly as it also gives information on therapeutic dosage and toxicity. A key factor in this model's burgeoning use^[51]^ is that benchmarking studies show that it provides results that are comparable to traditional in vivo models,[^52^, ^53^] such as mice, but it is more ethically compliant with the 3R's principle.^[54]^ Furthermore, as its complete genome sequence is now available,^[55]^ immune system mapping facilitating a detailed molecular understanding of host responses is possible.

Given the issues discussed above, it is perhaps unsurprising that studies involving metal complexes as novel antimicrobial leads have attracted increasing attention.[^56^, ^57^] Yet, although polypyridyl Ru^II^ complexes have been extensively studied as imaging probes[^58^, ^59^, ^60^, ^61^, ^62^, ^63^, ^64^] and anticancer therapeutics,[^65^, ^66^, ^67^, ^68^, ^69^] and the fact that as early as the 1950s the Dwyer group had demonstrated that [Ru(phen)_3_]^2+^ (phen=1,10‐phenanthroline) and its methylated derivatives were active against a range of Gram‐positive bacteria,[^70^, ^71^] apart for a few notable exceptions,[^72^, ^73^, ^74^] the potential of this class of compounds as antimicrobials has only just recently begun to be explored more widely.^[75]^

Also more recently, studies in the *G. mellonella* have begun to emerge. In 2019, Ude, et al. used *G. mellonella* to investigate the toxicity of Cu^II^ complexes that display activity against Gram positive pathogens,^[76]^ while Güntzel and colleagues demonstrated that treatment with CO releasing Mn^I^ complexes improve the survival rate of *Galleria* infected with *A baumannii* and (to a lesser extent) *P aeruginosa*.^[77]^ Very recently, O'Shaughnessy, et al. have shown that phenanthroline complexes containing Cu^II^, Mn^II^ and Ag^I^ centers can potentiate the effect of the conventional antibiotic gentamycin and reduce mortality rate in *G. mellonella* infected with therapeutically resistant *P. aeruginosa*.^[78]^

**Figure 1:**
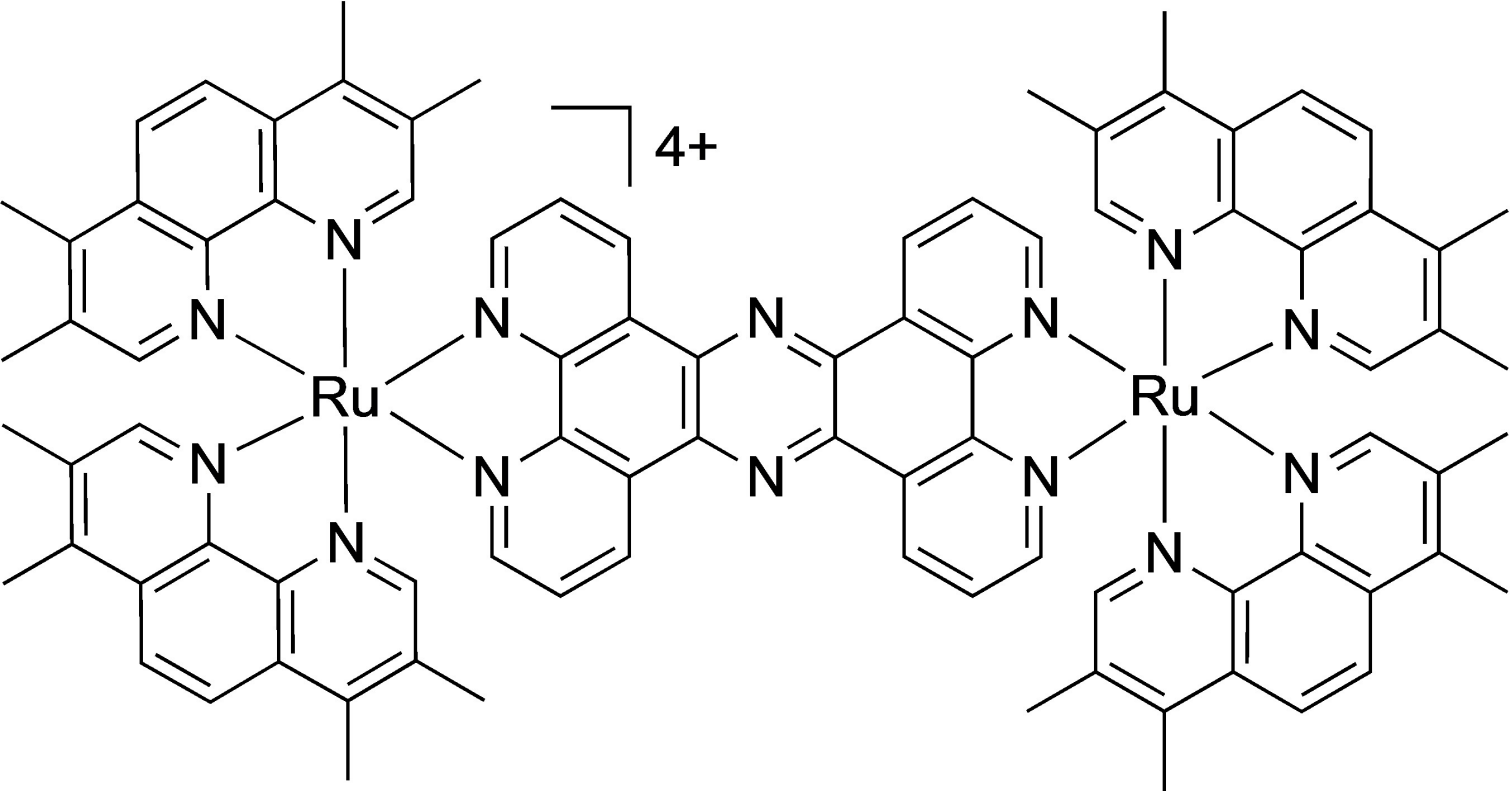
Structure of the cationic polypyridyl Ru^II^ complex, **1** ^4+^, studied in this report.

In this context – and as part of a program to develop new therapeutics[^79^, ^80^, ^81^, ^82^] and phototherapeutics[^83^, ^84^, ^85^, ^86^, ^87^] based on luminescent metal complexes – the Thomas group has previous reported on several antimicrobial leads including a dinuclear Ru^II^ compound, [**1**]Cl_4_, Figure  1, that displays high therapeutic activity against a range of Gram‐negative bacteria, including pathogenic multi‐drug resistant strains such as *E. coli* EC958,[^88^, ^89^, ^90^] and is also active on resistant Gram‐positive bacteria like *Staphylococcus aureus*.^[91]^

One of the attractions of exploiting such systems is that they are genuine theranostics. As [**1**]Cl_4_ is luminescent its internalization can be directly visualized through optical microscopy and the incorporation of two electron‐dense metal ions means it is also an excellent contrast probe for transmission electron microscopy, TEM.[^88^, ^91^] These intrinsic imaging properties, facilitated the identification of the action mechanism of the lead. Super resolution STED nanoscopy imaging, TEM, and membrane damage assays all confirmed that the complex disrupts the bacterial membrane structure before internalization, where it then binds bacterial DNA.[^88^, ^91^]

A subsequent transcriptomics‐based analysis confirmed that a pathogenic AMR *E coli* strain (EC958) exposed to **1** ^4+^ displayed downregulation of genes involved in membrane transport, but increased activity of an outer membrane repair mechanism.^[90]^

As in vitro and in vivo studies revealed that the complex is not toxic to eukaryotes – even at concentrations that are several orders of magnitude higher than its minimum inhibitory concentration in AMR pathogens^[88]^ – we set out to investigate its therapeutic potential in an standard infection model.

In this report, the in vitro potency of [**1**]Cl_4_ against a number of highly pathogenic strains of both *P. aeruginosa* and *A. baumannii* is explored and *G. mellonella* is used as a model to investigate its in vivo efficacy in the treatment of *A. baumannii*. In these latter studies, the optical and transmission electron microscopy imaging properties of [**1**]Cl_4_ were exploited to visualize bacterial clearance and confirm that a single dose of the complex totally irradicates the pathogenic infection in all treated larvae without any detectable deleterious effects to the *Galleria*.

## Results and Discussion

### Assessing MIC, MBC, and localization

Cation complex **1** ^4+^ was synthesized as a hexafluorophosphate salt through a reported procedure^[88]^ and was studied as its water‐soluble chloride salt, which was obtained via anion metathesis.

Our previous work revealed that – in contrast to most antimicrobials, including Ru^II^ systems[^72^, ^73^, ^74^] – the complex exhibits higher activity over Gram‐negative bacteria, such as an uropathogenic *E. coli* strain, than the Gram‐positive species *E. faecalis* and *S. aureus*.[^88^, ^91^] In these studies, we built upon previous results, and investigated five pathogenic strains of two different Gram‐negative ESKAPE bacteria. As both bacteria have common strains that exhibit carbapenem‐resistance they are in the WHO's list of Priority 1 – CRITICAL antibiotic‐resistant ‘priority pathogens’: for research and development.^[18]^

*A. baumannii* strains (AB12, AB16, AB184 and AB210) were chosen as they represent currently important clonal groups in the UK.[^92^, ^93^] All strains have been shown to exhibit multi‐drug resistance, including carbapenem. Additionally, two carbapenem resistant clinical‐isolate strains of *P. aeruginosa*, from Public Health England: PA1‐PA_ 007_ IMP (IMP‐metallo β‐lactamase producing) and PA2‐PA_ 004_ CRCN (carbapenem and cephalosporin resistant), as well as a pan‐drug resistant clinical isolate strain (PA2017) from the University of Surrey were tested.

The minimum inhibitory concentrations, MIC, of the complex were obtained in both glucose defined minimal media (GDMM) and nutrient rich Mueller‐Hinton‐II (MH‐II). Both media have been used in antimicrobial reports on metal complexes – but MH‐II is the medium recommended by the European Committee on Antimicrobial Susceptibility Testing^[94]^ and it more closely replicates the conditions the bacteria will experience in the *G. Mellonella*. As with previous studies, the complex exhibited a higher activity in GDMM, however comparable activities in MH‐II were observed – Table 1.

**Table 1:** MIC (μM) results for [**1**]Cl_4_ treatment of *A. baumannii* pathogenic (AB12, AB16, AB184, AB210) strains and *P. aureginosa* pathogenic (PA2017) strain in GDMM and MH‐II. Data averaged from 3 biological repeats.

AB12	AB16	AB184	AB210	PA1	PA2	PA2017
GDMM values/μM						
0.6	1.0	0.60	0.50	4.8	4.8	9.57

MH‐II values/μM						
2.8	1.6	2.4	1.6	9.6	9.6	19.1

Strikingly, the MIC values are very low for all the *A. baumannii* strains. While comparative values are higher for *P. aeruginosa*, it is notoriously difficult to treat pathogen as it exhibits an innate resistance to a wide range of antibiotics, partly due to the cells membranes hosting several multidrug efflux pumps.^[21][95]^ Notably, **1** ^4+^ continues to exhibit potent activity in the *P. aeruginosa* strains exhibiting carbapenem resistance. As far as we are aware, this is the first inert Ru^II^ polypyridyl complex to exhibit activities comparable to clinical antibiotics on *any P. aeruginosa* strain.

Estimates of minimum bactericidal concentrations, MBC, for **1** ^4+^ were also obtained and are summarized in Table 2. The same increase in MBC values between GDMM and MH‐II is observed as for the MIC. Again a lowered potency is observed on the *P. aeruginosa* strain.

**Table 2:** MBC (μM) results for [**1**]Cl_4_ treatment of *A. baumannii* pathogenic (AB12, AB16, AB184, AB210) strains and *P. aureginosa* pathogenic (PA2017) strain in GDMM and MH‐II. Data averaged from 3 biological repeats.

AB12	AB16	AB184	AB210	PA1	PA2	PA2017
GDMM values/μM						
1.2	2.4	1.6	0.8	9.6	9.6	14.4

MH‐II values/μM						
15.9	7.9	6.3	3.2	18	18	38.3

As antibacterial agents are usually considered bactericidal if the MBC/MIC ratio is no more than four the data summarized in Tables 1 and 2 indicate that **1** ^4+^ is bactericidal across all strains of these two pathogens in both media, causing a ≥99.9 % reduction in the viability of the initial bacterial inoculum.

**Figure 2:**
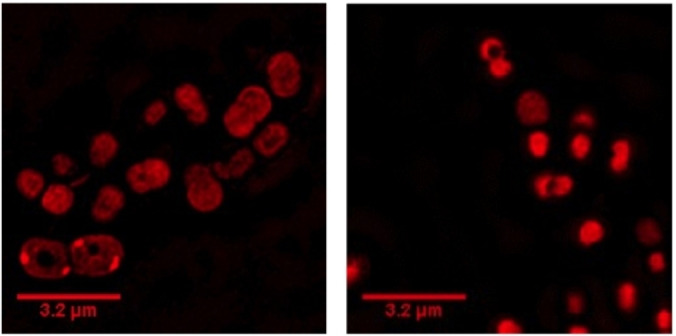
Localization of **1^4+^** in *A. baumannii* AB184 cells visualized through SIM at 10 min (left) and 60 min (right). Complex **1** ^4+^ excited at 450 nm using the A568 filter. After treatment with 0.8 μM **1** ^4+^, cells were washed with PBS before fixing with paraformaldehyde (4 %).

The lineage of AB184 was initially associated with casualties returning from Iraq conflict and as a consequence it is a very common *A. baumannii* clonal group in both the UK and the USA.^[96]^ As it was observed that **1** ^4+^ displayed high activity against AB184, the luminescent properties of the complex were used to investigate uptake by this strain through super‐resolution, structured illumination microscopy (SIM), which allows for sub‐diffraction limited resolutions of ∼100 nm – Figure 2.

The uptake and localization behavior in *A. baumannii* was entirely consistent with our previous detailed studies.[^88^, ^90^, ^91^] Complex **1** ^4+^ initially binds to the membrane of cells; which is again consistent with binding to anionic lipopolysaccharides embedded within the membrane.^[88]^ At 60 minutes, luminescence from **1** ^4+^ is no longer observed from the outer membrane; instead, the compound has internalized, likely binding to DNA as it does in other Gram‐negative pathogens such as the EC958 strain of *E coli*.^[88]^ Indeed, these observations are consistent with those obtained with other cationic species which bind to glycerophospholipids that make‐up the inner membrane of *A. baumannii* cells.^[97]^

It is known that some Ru^II^ complexes can function as photoactivated antimicrobials^[98]^ most often through the well‐delineated mechanism of singlet oxygen sensitization.[^75^, ^99^] However, although the bacterial studies were carried out in the dark, we saw no evidence that exposure to light increased toxicity effects. Indeed, when singlet oxygen quantum yields were directly measured by assessing luminescence at 1270 nm following photoexcitation of [**1**](PF_6_)_4_ in acetonitrile a ϕ(^1^O_2_) estimates of only 10 % was obtained.

As previous studies have determined the toxicity of **1** ^4+^ against a representative non‐cancerous human cell‐line, HEK293 to be IC_50_=135 μM,^[88]^ therapeutic indices for the compound against all the *A. baumannii* strains and *P. aeruginosa* were determined – Table 3. Although the therapeutic index is high across all the pathogenic strains, it is significantly higher for the *A. baumannii* strains, which reflects its higher potency against this pathogen, indicating that the compound could be a particularly effective treatment for *A. baumannii* infections.

**Table 3:** Therapeutic index: IC_50_/MIC ratio for all strains of bacteria in GDMM.

AB12	AB16	AB184	AB210	PA1	PA2	PA2017
225	135	225	270	28.1	28.1	14.1

### Bacterial infection screen

With the aim of assessing whether the in vitro activity of **1** ^4+^ against AMR strains is carried through into an in vivo model, the potential of employing *G. mellonella* as an infection model for these multidrug resistant pathogens was first explored. Again, AB184 was chosen for this study as it is a representative MDR strain of *A. baumannii* and the highly resistant PA2017 strain was chosen as the *P. aeruginosa* strain.

In these experiments, it was found that PA2017 at concentrations of 10^3^ CFU/mL or above killed 100 % of all inoculated *G. mellonella* within 24 h – see Supporting Information; Figures S1–3. In fact, this strain is so virulent that a reliable and statistically valid infection model could not be developed, even at very low concentrations. Therefore, the infection model was carried forward solely with AB184 – Si; Figures S1, S4 and S5.

In developing the model, *G. mellonella* activity over 120 h was scored at 0–4 (0: no movement, 1: minimal movement, 2: movement on stimulation, 3: movement without stimulation) and melanization was scored between 0–3. There is extra scoring for cocoon formation as evidence of cocoon formation was observed in the non‐injected controls at the 120‐hour time‐point. Additionally, at each time‐point, the concentration of bacteria within the larval hemolymph was determined via CFU/mL counts after extractions.

These experiments revealed that AB184 causes a detectable infection in hemolymph. As there is no decrease in colony forming units over time this infection is pathogenic, with larvae being unable to clear the infection using their innate immune system. This indicates that, in these conditions, AB184 is suitable to be employed in an *G. mellonella* infection model as any decrease in CFU/mL with treatment with an antimicrobial compound can be attributed to the effects of the compound, and not larvae's immune system.

Before investigating treatment regimes, studies confirmed our original report that **1** ^4+^ was non‐toxic to *G. mellonella* up to concentrations of at least 80 mg/kg (Si, Figures S6 and S7), the average maximum daily dose for a clinical antibiotic.^[88]^ Therefore, in the final infection model, larvae were injected with AB184 at concentrations of 10^5^ or 10^6^ CFU/mL, then 30 minutes later with **1** ^4+^ at 40 or 80 mg/kg and then incubated in the dark. The larvae were then monitored over 120‐hours and scored using the previously delineated scheme. Survival curves were plotted for AB184‐infected larvae treated with **1** ^4+^, alongside water and AB184 only controls.

For both AB184 control concentrations (10^5^, 10^6^ CFU/mL) Log‐Rank statistical t‐tests indicated a (**) difference in larvae survival between the water controls: P values 0.0048 and 0.0045 respectively. It's therefore determined that AB184 is pathogenic to *G. mellonella* at concentrations from 10^5^ CFU/mL upward. Significantly, survival curves for AB184 injected larvae at both 40 mg/kg and 80 mg/kg treated with **1** ^4+^ showed no significant difference to the water controls, indicating that the compound kills bacteria and clears the larval infection – for activity and melonization scores, see the Supporting Information Figures S8 and S9.

**Figure 3:**
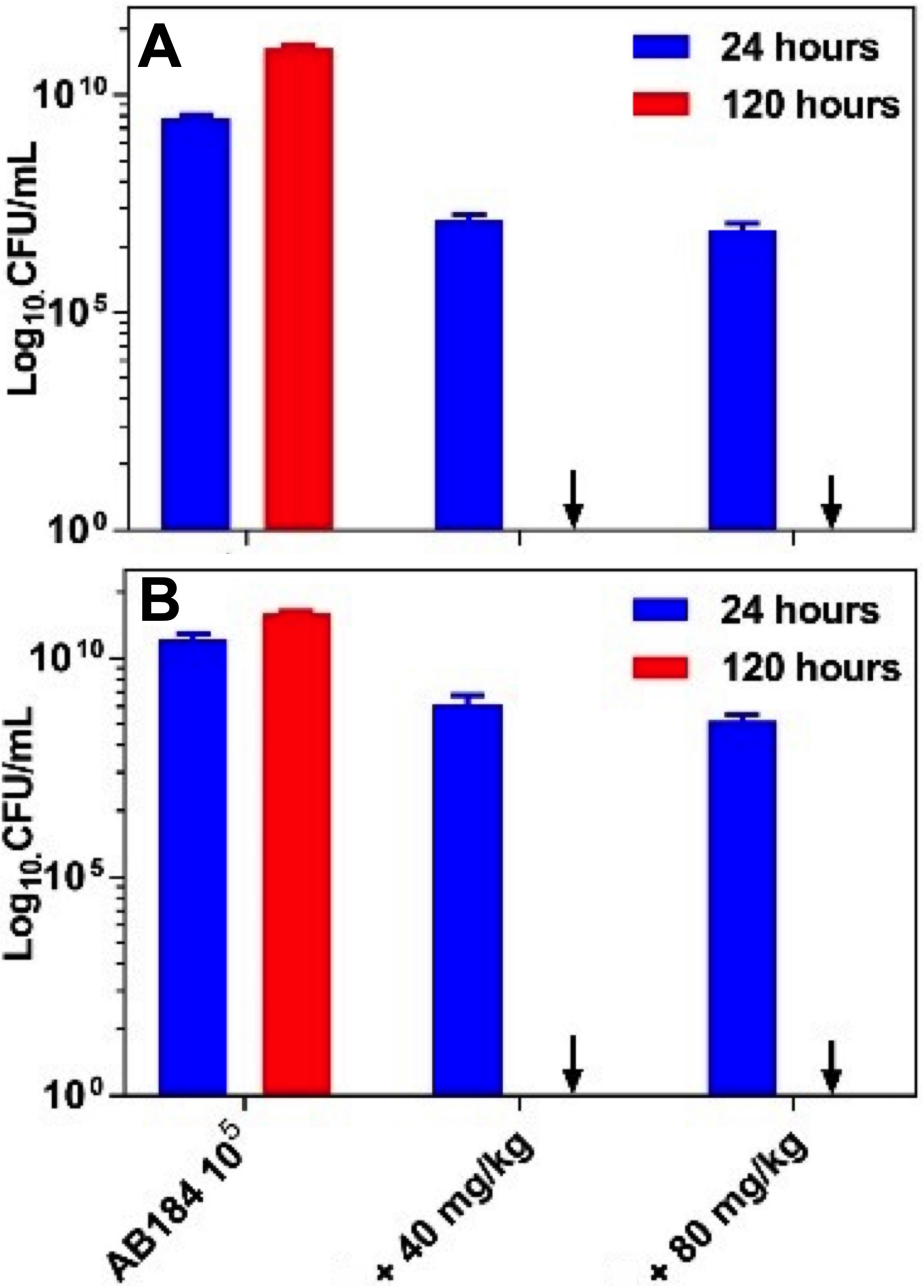
*Galleria mellonella* infection model. Colony forming unit counts from larvae hemolymph extractions Larvae were injected with: (A) 10^5^ CFU/mL of AB184 or (B) 10^6^ CFU/mL of AB184. In both cases, the *G. mellonella* were treated with **1^4+^** (40/80 mg/kg) and the results were compared to untreated larvae. Extractions were taken at 24 and 120 h. Protocol: Larvae were injected with bacteria in their right pro‐leg, then those that were treated received a dose of **1^4+^** 30 minutes later in their left pro‐leg. Larvae were incubated for 120 h at 37.5 °C. Error bars represent the results from three repeats.

To ensure that the observed difference between the survival of the co‐injected larvae and the bacteria injected controls was a result of the antibacterial action of **1** ^4+^, CFU/mL counts were conducted from extracted hemolymph – Figure 3. From 96 h onward, no bacterial colonies were formed confirming that the AB184 infection was completely eradicated by treatment with of **1** ^4+^ as a single dose at either of the tested concentrations.

### Using 1^4+^ to visualize bacterial infection and its clearance within hemolymph

**Figure 4:**
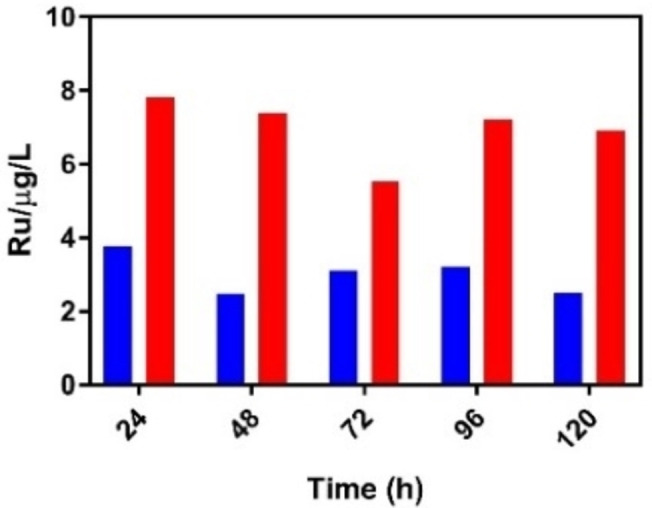
Ruthenium hemolymph content (μg/mL) measured by ICP‐AES. The hemolymph Ru content for *Galleria* injected with **1^4+^** 80 mg/kg (red) and 20 mg/kg (blue) Galleria were injected with 10 μL **1^4^** ^+^/water into their left pro‐leg, incubated at 37.5 °C. Ru content was determined through ICP‐AES after this period.

In controls before the infection experiments, it was noticed injection of the compound caused the larvae's hemolymph to luminesce red confirming its localization within the *G. mellonella* circulatory system. To quantitate this effect the amount of ruthenium in the larvae's hemolymph was assessed using ICP‐AES. Hemolymph was extracted from a small incision beneath the larvae's head and ICP‐AES experiments were used to monitor Ru content over the 120 h for larvae treated with 20 and 80 mg/kg of **1** ^4+^ – Figure 4.

**Figure 5:**
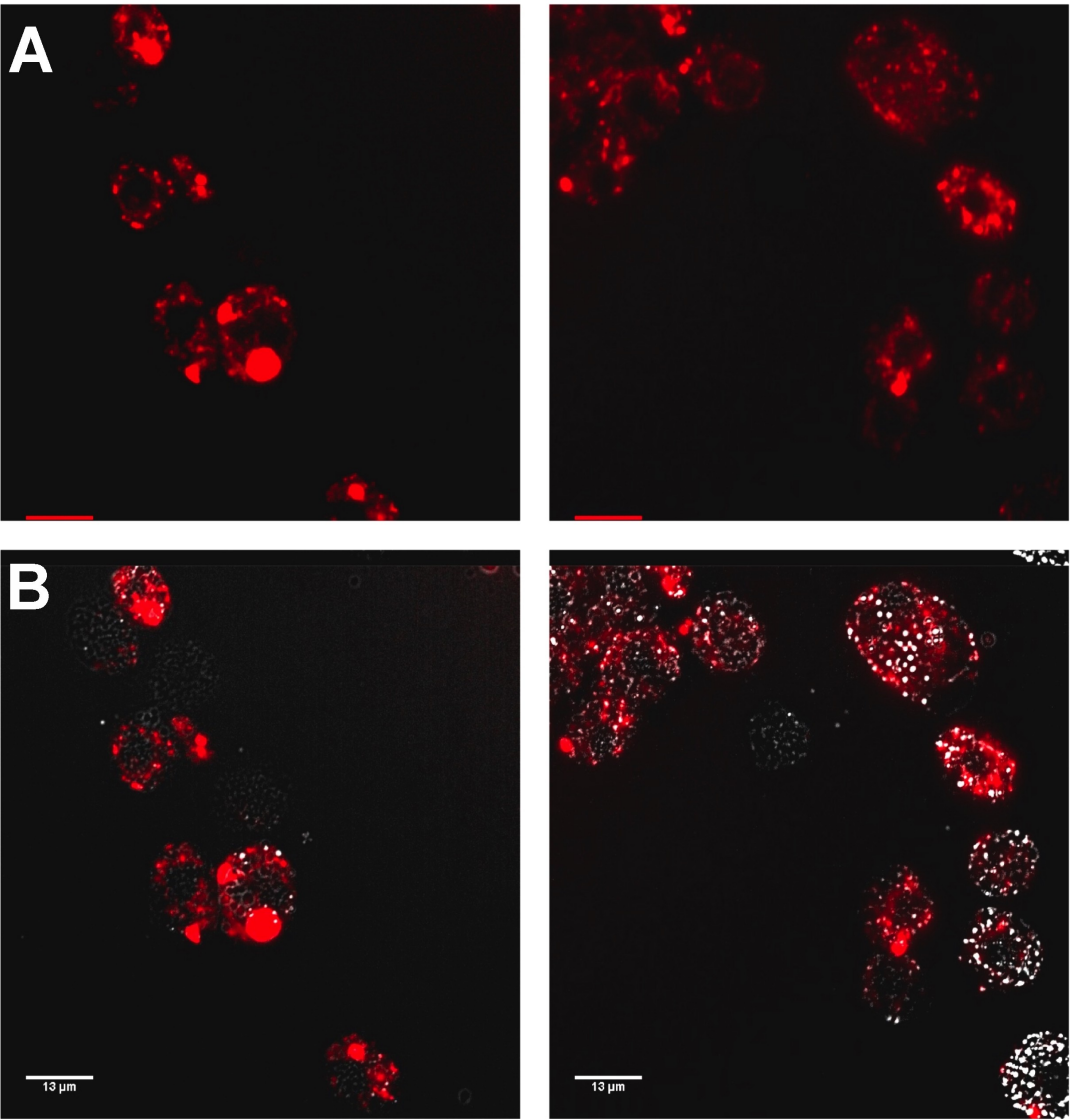
Confocal microscope images confirm localization of **1^4+^** in A*. baumannii* AB184 cells within the larvae's hemolymph. A: Selected cells images using the emission of **1** ^4+^ on excitation at 450 nm using A568 filter. B: Combined phase contrast/emission image. Extracted hemolymph cells were washed with nitric acid before fixing with paraformaldehyde (16 %).

Given that **1** ^4+^ concentrations are high in the hemolymph and – as illustrated by Figure 2 – the complex is taken up and images *A. baumannii* cells in vitro, the possibility of *directly* monitoring infection clearance through optical microscopy was investigated. Hemolymph from live larvae was extracted 24‐hours after infection and treatment with **1** ^4+^. Extractions were performed under anaesthetized conditions and bacteria cells were imaged using confocal microscopy – Figure 5. The images confirm that once injected into larvae, **1** ^4+^ preferentially localizes in the bacteria cells. This experiment also revealed an interesting phenomenon.

As complex **1** ^4+^ incorporates two electron dense ruthenium centers it is also an excellent contrast stain for transmission electron microscopy (TEM),[^100^, ^101^, ^102^] therefore we also investigated if the clearance of the *A. baumannii* infection could also be monitored through this technique.

**Figure 6:**
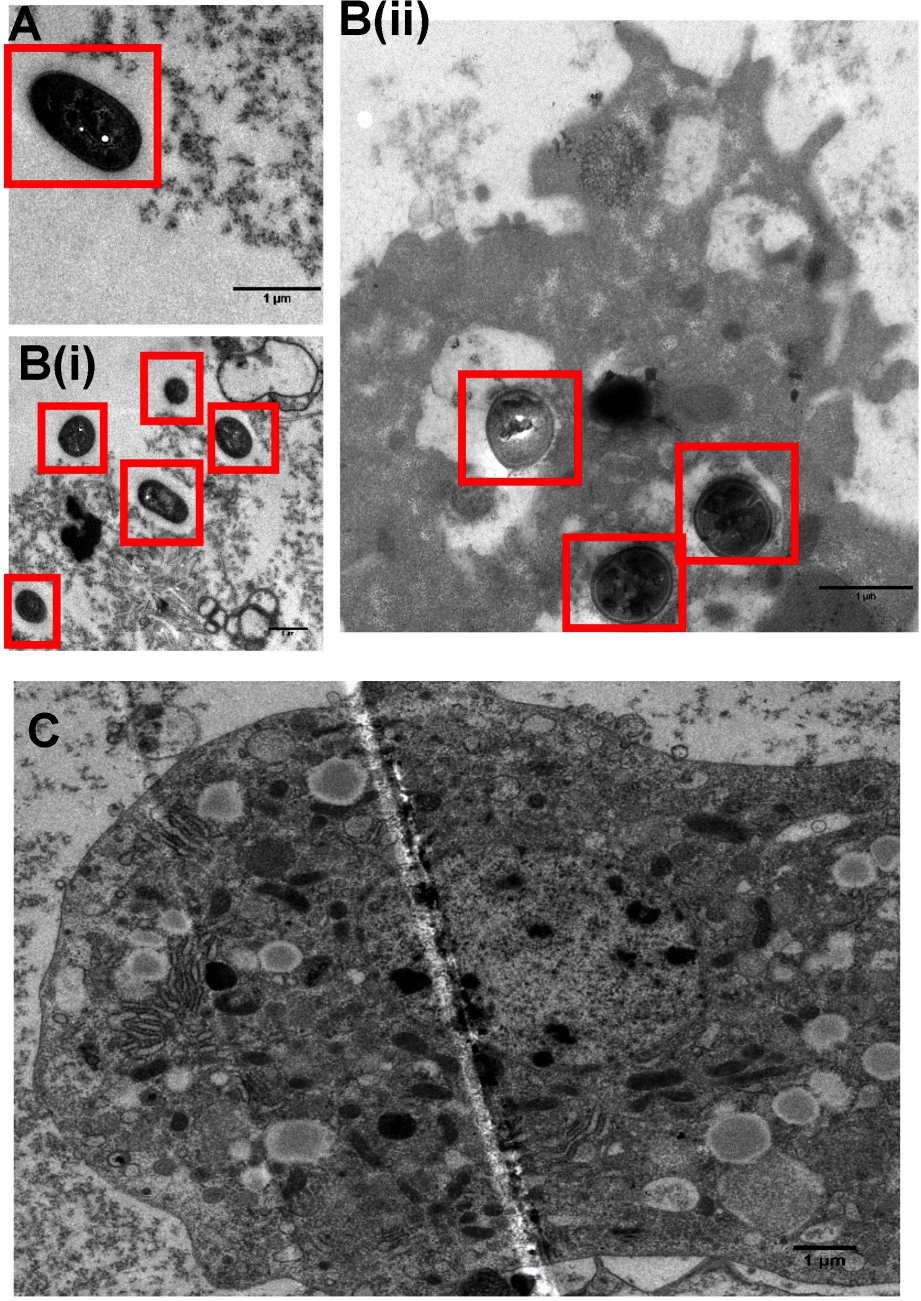
TEM images showing **1^4+^** in A*. baumannii* AB184 cells within the larvae's hemolymph at 24 (A), and 48 (B) Highlighted images of hemolmpyh (BI) and hemocyte cells (BII) at 48 h. (C) At 96 h no bacteria were observed in the extracted hemolymph cells. Cells were fixed with 2.5 % glutaraldehyde in sodium cacodylate buffer (pH 7.4).

Hemolymph from infected larvae was extracted at 24, 48 and 96 h post treatment and imaged through TEM using **1** ^4+^ as the sole contrast stain – Figure 6.

The images clearly reveal that there are still intact *A. baumannii* cells within the extracted hemolymph at 24 h. However, while live and dead *A. baumannii* cells were observed at 48 h in both the hemolymph and in hemocyte cells,by 96‐hours there were no bacteria observed within either the hemolymph and hemocyte cells. Strikingly, the hemocyte cells were still intact with no visible damage. As samples were only stained with **1** ^4+^; and sites of contrast arise from binding of the compound, these experiments offer further evidence that **1** ^4+^ is taken up by *A. baumannii* but also reveal that, like previously reported analogues,[^86^, ^102^] it appears to bind to mitochondria and lysosomes within the hemocyte cells.

Taken together, the optical microscopy and TEM images are consistent with the results from the CFU hemolymph extraction assays and live/dead scoring, confirming a single dose treatment of **1** ^4+^ results in a total clearance of a pathogenic *A. baumannii* infection of *Galleria* larvae within 96 h.

## Conclusions

Our in vitro experiments show that antimicrobial lead **1** ^4+^ is active against resistant strains of *A. baumannii* and – albeit to a lesser extent – *P. aeruginosa*. Together, these bacteria present some of the greatest global threats to health and are two of the most resistant organisms encountered in clinical practice; *A. baumannii* alone, has been estimated to cause one million infections a year, with carbapenems resistance rates being as high as >95 %. The need to develop new therapeutic leads against these pathogens is particularly urgent as strains that are resistant to the last‐line antibiotics polymyxins and tigecycline are emerging.

Significantly, the complex also cleared an infection of a multidrug resistant *A. baumannii* strain within *G. mellonella* in a single dose. In terms of treatment two key observations arise from the infection model study: first, the infection was cleared in all treated larvae, even at the lowest doses of **1** ^4+^; second, the infection was successfully treated at concentrations of the complex that produced no detectable toxicity effects in *G. mellonella*. Furthermore, the fact that **1** ^4+^ is intrinsically luminescent and taken up by the infecting bacteria means it is a genuine theranostic as the larval hemolymph infection could be directly monitored until the host was clear of infection.

This is the first time any single agent combining imaging properties capable of monitoring an infective agent *and* the ability to clear a highly resistant ESKAPE infection in an in vivo model with a single dose has been reported. This study underlines the potential metal complexes offer as new and novel therapeutics, particularly as Ru^II^ complexes have been very recently shown to offer promise as potential leads against pernicious *mycobacter* pathogens,^[103]^ Further studies in *G. mellonella* and murine models aimed at optimizing the therapeutic properties of **1** ^4+^ and derivatives, and identifying new leads are currently underway.

## Conflict of interest

The authors declare no conflict of interest.

1

## Supporting information

As a service to our authors and readers, this journal provides supporting information supplied by the authors. Such materials are peer reviewed and may be re‐organized for online delivery, but are not copy‐edited or typeset. Technical support issues arising from supporting information (other than missing files) should be addressed to the authors.

Supporting Information

## Data Availability

The data that support the findings of this study are available on request from the corresponding author. The data are not publicly available due to privacy or ethical restrictions.
