# Supplementary material for: Clearing an ESKAPE Pathogen in a Model Organism; A Polypyridyl Ruthenium(II) Complex Theranostic that Treats a Resistant Acinetobacter baumannii Infection in Galleria mellonella
Source: Chemistry. 2023 Jan 12;29(11):e202203555. doi: 10.1002/chem.202203555 (PMC10946903; doi:10.1002/chem.202203555)
Supplement: Supplementary file 1 — Supporting Information [file CHEM-29-0-s001.pdf]

Chemistry–A European Journal

Supporting Information

Clearing an ESKAPE Pathogen in a Model Organism; A Polypyridyl Ruthenium(II) Complex Theranostic that Treats a Resistant Acinetobacter baumannii Infection in Galleria mellonella

Kirsty Smitten, Hannah M Southam, Simon Fairbanks, Arthur Graf, Adrien Chauvet, and Jim A Thomas*

S1. Chemistry methods

S1a. [Ru(3,4,7,8-tetramethyl-1,10-phenanthroline)$_2$Cl$_2$]$^{2+}$

S1b. [{Ru(TMP)$_2$}$_2$(tpphz)](PF$_6$)$_4$, [1](PF$_6$)$_4$

S1c. Singlet Oxygen Quantum Yield

S1d. Anion Metathesis

S2. Microbiology methods

S2a. Bacterial strain information and general growth procedures

S2b. Determination of minimum inhibitory concentrations (MICs) and minimum bactericidal concentrations (MBCs)

S2c. Structured illumination microscopy

S2d. Hemolymph ruthenium accumulation

S2e. Bacterial infection screen

S2f. Bacteria infection model

S2g. *Galleria* hemolymph light microscopy imaging

S2h. *Galleria* hemolymph transmission electron microscopy (TEM)

S3. Supplementary data

S3a. Kaplan Meier survival curves for AB184 and PA2017 10^6-10^8 CFU/mL

S3b. Images of PA2017 larvae injected with 10^3 CFU/mL

S3c. PA2017 percentage activity and melanisation

S3d. Larvae images at 120 hours for larvae infected with AB184 10^2 - 10^5 CFU/mL

S3e. AB184 percentage activity and melanisation

S3f. Compound only controls infection model

S3g. Larvae images from initial tox screen ½ MIC, MIC and 2MIC concentrations of 1^{4+}

S3h. Activity/melanisation graphs for AB184 10^6 CFU/mL infection mode

S3i. Kaplan Meier survival curves for AB184 infection model and treatment with 1^{4+}

References

APPENDIX: NMR and ESI-MS of complex used in studies.

Supplementary 1 – Chemistry methods

S1a. [Ru(3,4,7,8-tetramethyl-1,10-phenanthroline)$_2$Cl$_2$]$^{2+}$

RuCl$_3$.3H$_2$O (1.14 g, 5.50 mmol), TMP (2.4 g, 10.16 mmol) and LiCl (1.47 g, 34.68 mmol) were heated for 8 h under reflux in DMF (19 mL). The reaction mixture was cooled to room temperature and acetone was added (100 mL). The solution was stored at 4 °C for 16 h forming a dark purple precipitate. The product was washed with water and ethanol and dried *in vacuo*. Mass = 2.07 g (3.21 mmol, 63.2 %) purple solid. MS m/z (%): 609.1 (62) [M −Cl]+, 637.1 (100) [M]+ 667.1 (44) [M + Na]+. Carbon monoxide displaced one of the chlorines.

$[M-3(PF_6)]^{3+}$. ^1H NMR (MeCN-d_6) δ (splitting integration): 2.1 (s, 48H), 7.8 (s, 4H), 7.9 (t, 8H), 8.2 (dd, 4H), 8.4 (s, 8H)

S1b. [{Ru(TMP)$_2$}$_2$(tpphz)](PF$_6$)$_4$, [1](PF$_6$)$_4$

$Ru(TMP)_2Cl_2]^{2+}$ (1.12 g, 1.73 mmol) and tpphz (0.260 g, 0.68 mmol) were added to a 1:1 solution of ethanol and water (80 mL). The solution was refluxed for 12 h under argon. After completion the reaction mixture was cooled to room temperature and stored at 4 °C for 16 h. The red solution was filtered, and ethanol removed by rotary evaporation. A saturating amount of NH_4PF_6 was added; this caused the formation of a dark red precipitate. The precipitate was collected *via* vacuum filtration, washed with water and recrystallised in acetonitrile by addition of diethyl ether. The product was dried *in vacuo* and purified on an alumina column, solvent system: 95 % MeCN, 3 % dH_2O and 2 % KNO3. Mass = 1.22 g (0.58 mmol, 85.7 % yield). MS; m/z (%): 911 (10) [M − 2(PF6)]2+, 559 (100) $[M-3(PF_6)]^{3+}$. ^1H NMR (MeCN-d_6) δ (splitting integration): 2.1 (s, 48H), 7.8 (s, 4H), 7.9 (t, 8H), 8.2 (dd, 4H), 8.4 (s, 8H), 9.9 (dd, 4H). ^1H NMR (Acetone-d6) δ (splitting integration): 2.1 (dt, 48H), 8.0 (m, 4H), 8.1 (s, 4H), 8.2 (s, 4H), 8.52 (d, 4H), 8.6 (s, 8 H), 10.1 (d, 4H). Elemental analysis [{Ru(3, 4, 7, 8-Tetramethyl-1,10-phenanthroline)$_2$}$_2$(tpphz)](PF6)$_4$·5.5H2O, C88H87N14O5.5$Ru_2P_4F_{24}$ Calculated: C; 47.93, H; 3.97, N: 8.89. Found C; 47.92, H; 3.83, N; 8.82. Accurate mass analysis: C88H76N14[102Ru]$_2^{4+}$ Calculated 383.1111. Found 383.1112.

S1c Singlet oxygen quantum yield

The quantum yield of singlet oxygen generation on photo-excitation of [**1**]$(PF_6)_4$ was determined using a Q-Switch Nd: YAG laser (n= 355 nm, 8 nm pulse length, laser model LS-1231M from LOTISII) through directly monitored production of singlet oxygen is by its emission at ~1270nm. A high-contrast bandpass optical filter (1277 nm, 28 nm FWHM, by Izovac, Belarus) was placed in front of the InGaAs photodiode to selectively detected the singlet oxygen emission (J22D-M204-R03M-60-1.7, Judson Technologies). The output signal was recorded with a digital oscilloscope (TDS 3032B Tektronix). The samples and standard (phenalenone) were diluted to have the same optical density at 355 nm, to ensure that the same laser pulse energy yields the same number of molecules to be excited. Singlet oxygen values were acquired with increasing laser power, up to 60 mJ, to avoid saturation of the detector. A linear relationship between singlet oxygen signal amplitude (y-axis) and power (x-axis) was assumed. The quantum yield value is then obtained by dividing the sample fitted signal's slope by that of the standard.

S1d. Anion metathesis

The hexafluorophosphate salt of each complex was dissolved in the minimum volume of acetone, and a saturated solution of tetrabutylammonium chloride in acetone added. The resultant precipitated chloride salt was collected by filtration, washed with cold acetone, and dried *in vacuo*.

Supplementary 2 – Microbiology methods

S2a. Bacterial strain information and general growth procedures

Microbiological studies were conducted with a pathogenic, clinical isolate strain of *Pseudomonas aeruginosa,* PA2017. The clinical isolate was retained from a patient at The University of Surrey. Four clinical isolate strains of *Acetinobacter baumannii* were studied: AB12, AB16, AB184 and AB210.[1-4] These strains were chosen as they represent the most prevalent clonal groups. Bacteria were routinely grown under septic, aerobic condition in autoclave-sterilised culture medium at 37 °C. Lysogeny Broth (LB) (Formedium), Mueller-Hinton II (MH-II) (Sigma-Aldrich) and Brain-Heart Infusion (BHI) (Sigma-Aldrich) were prepared as per manufacturers' instructions. Glucose defined minimal medium (GDMM) was prepared by dissolution of 4 g/L K_2PO_4,1 g/L KH_2PO_4,1 g/L H_4Cl, 10 mg/L $CaCl_2$ and 2.6 g/L K_2SO_4 in deionized H_2O with supplementation of 10 mL/L Trace Elements solution. The pH was adjusted to 7.4 by the addition of NaOH. GDMM was sterilized by autoclaving and then further supplemented with 1 mM $MgCl_2$ and 20 mM glucose prior to growth studies. Trace elements contained 5 g/L ethylenediaminetetraacetic acid (EDTA), 0.5 g/L $Fe(III)Cl_3·6H_2O$, 50 mg/L ZnO, 10 mg/L $CuCl_2·2H_2O$, 10 mg/L $CoNO_3·6H_2O$, 10 mg/L H_3BO_3, 0.12 mg/L $(NH_4)2MoO_4$ and 17 mg/Na_2O_4Se.

Prior to experiments, bacterial starter culture were prepared by inoculating LB with a single bacteria colony and then grown overnight at 37 °C with shaking for 16−18 h. Starter cultures were washed once and resuspended in the appropriate growth medium for each experiment. For short-term storage, bacterial stocks

were maintained on nutrient agar plates at 4 °C for 2−3 weeks. For long-term storage, strains were stored as cell suspensions in 30% (v/v) LB 70% (w/v) glycerol at −70 °C.

S2b. Determination of minimum inhibitory concentrations (MICs) and minimum bactericidal concentrations (MBCs)

MICs and MBCs of $\mathbf{1}^{4+}$ were determined *via* the standard broth-dilution method in 96-well microtiter plates in either MH-II, as recommended by European Committee of Antimicrobial Susceptibility Testing (EUCAST) or in GDMM. The MIC was evaluated using 2-fold increasing concentrations of $\mathbf{1}^{4+}$ between 2 to 512 μgmL^{-1} against a bacterial inoculum of 10^7−10^9 colony forming units per mL (CFU mL^{-1}), corresponding to an optical density at 600 nm (OD600) of 0.05−0.075. Plates were incubated at 37 °C for 20 h. After this time, the level of turbidity in each well was used to determine the extent bacterial cell growth in the presence of $\mathbf{1}^{4+}$. The minimal concentration of compound that did not permit bacterial growth was determined to be the MIC. For MBC determination, 10 μL samples of each well were then transferred to nutrient agar plates and further incubated at 37 °C. The lowest concentration of compound in which no CFU were observed after plating was determined to be the MBC. All MIC and MBC experiments were done with 3 biological independent repeats all conducted in triplicate.

S2c. Structured illumination microscopy

An overnight culture of *A. baumannii,* AB184, was grown in LB. The cells were washed (×2) and resuspended in GDMM. The cells were diluted to OD_{600} 0.05 and grown to early exponential phase (OD 0.3−0.4) in GDMM (50 mL). One mL of culture was harvested, and the compound added at MIC concentration. One mL cultures were then harvested at 10 and 60 min. The bacteria were pelleted (centrifuge, 14 000 rpm, 90 s), and supernatant removed. The pellet was suspended in fixant (4 % paraformaldehyde in PBS, 1 mL) and placed on a rotary wheel at room temperature for 30 min. The samples were washed in PBS and frozen as a pellet**.** Slide coverslips were sonicated in 1 M KOH for 15 min, then coated in polylysine solution for 30 min. The pellets were suspended in 5 μL of a SlowFade Gold Antifade Mountant (Thermo Fisher). The suspension was mounted onto the slide and the coverslip placed on top. Slides were imaged using the structured illumination (SIM) microscope. Imaging was done using the 1514 immersion oil and mol_probes microscope setting with a 450 nm excitation laser and the A568 emission filter. OMX SI reconstruction was performed on images and images were processed and analyzed using FIJI ImageJ software with the SIMCheck plugin to perform a 16-bit conversion.

S2d. Hemolymph ruthenium accumulation

Accumulation of 1^{4+} within the larvae's hemolymph was determined by measuring hemolymph metal content by inductively coupled plasma-atomic emission spectroscopy (ICP-AES). Larvae were injected with 20 and 80 mg/kg of 1^{4+} (10 μL) using a 1 mL Hamilton Syringe into their left pro-leg. The larvae were incubated at 37.5 °C for 120 hours. Every 24 hours the hemolymph was extracted from a larvae for each concentration by creating a small incision under the larvae's head. Incisions were made using a surgical knife or hair dressing scissors both were sterilized prior to incision using ethanol. Samples were centrifuged at 5000g for 20 min at 4 °C to obtain hemolymph cell pellets and the supernatant containing unbound extracellular 1^{4+} was discarded. The resultant cell pellets were then washed twice in 0.5% (v/v) Aristar nitric acid to remove loosely bound residual 1^{4+}. To prepare cell material for ICP-AES, cell pellets were resuspended in 0.5 mL Aristar nitric acid (69% (w/v) and then placed in a sonicator bath for 30 min to completely dissolve cells. The resulting digest was then diluted to a final volume of 5 mL with diluted nitric acid, and then samples were analyzed on a SpectroCirosCCD (Spectro Analysis) inductively-coupled plasma-atomic emission spectrophotometer. Levels of Ru in the samples were determined by a calibration curve using multielement standard solutions containing 0.1, 0.2, 5, and 10 mgL^{-1} Ru**.**

S2e. Bacterial infection screen

TruLarv Galleria mellonella were used for this study to ensure they were reared without antibioticsand were all a similar weight. For the controls and each bacteria concentration 14 *Galleria* larvae were used. Insects were injected on the initial day with 10 μL of *A. baumannii* or *P. aeruginosa* (10^1-10^3 CFU/mL). Initial experiments were also conducted using 10^5-10^8 CFU/mL. Once injected larvae were incubated, in a petri-dish containing filter paper, at at 37.5 °C. Three analysis tests were conducted at 0, 24, 48, 72, 96, and 120 h. Activity scores were recorded: 0, no movement; 1, corrects itself; 2, movement on stimulation; 3, movement without stimulation. Live/dead scores recorded to produce percentage survival curves. Melanization was scored on a scale of 0−4: 0, completely black; 1, black spots; 2, tail/ line black; and 4, none. At each time-

point 1 larvae per condition was sacrificed and the hemolymph extracted. Mantel-cox log rank statistical t-tests were used to determine the significant difference between the controls and the AB184 treated larvae. The neat hemolymph was serial diluted down (1:10) in PBS. Serial dilutions were plated onto nutrient agar in triplicate and incubated, inverted, overnight at 37.5 °C. Bacteria colonies were counted to produce bacterial growth curves from the larvae's hemolymph. Once the experiment was finished larvae were disposed of in a humane manner.

S2f. Bacteria infection model

For each condition 14 *Galleria* larvae were used except for the compound only control (with no larvae sacrifice) where 8 larvae were used. The following protocol was followed for each condition: +ve control - larvae were injected with 10 μL of *A. baumannii* AB184 (10^5/10^6 CFU/mL) into their right pro-leg; -ve control – larvae were injected with 10 μL MilliQ water into their left pro-leg; $\mathbf{1}^{4+}$ control – larvae were injected with 10 μL of $\mathbf{1}^{4+}$(40/80 mg/kg) into their left pro-leg; $\mathbf{1}^{4+}$ and AB184 - larvae were injected with 10 μL of *A. baumannii* AB184 (10^5/10^6 CFU/mL) into their right pro-leg, 30 minutes later – larvae were injected with 10 μL of $\mathbf{1}^{4+}$(40/80 mg/kg) into their left pro-leg. mL. Once injected larvae were incubated, in a petri-dish containing filter paper, at 37.5 °C. Larvae were assessed at 0, 24, 48, 72, 96 and 120 h using the three analysis tests discussed above. Statistical t-tests were used to determine significant differences between the conditions. As with previous hemolymph extractions were performed to monitor the clearance of the infection. Hemolymph extractions were performed on 1 larvae per condition every 24 hours except for the $\mathbf{1}^{4+}$ only controls.

S2g. *Galleria* hemolymph light microscopy imaging

From the above experiment at 24-hours hemolymph was extracted from 1 larvae in the $\mathbf{1}^{4+}$ + AB184 (80 mg/kg, 10^6 CFU/mL condition). Cells were pelleted (microfuge, 14 000 rpm, 90 s), and supernatant removed. The pellet was suspended in fixant (4 % paraformaldehyde in PBS, 1 mL) and placed on a rotary wheel at room temperature for 30 min. The samples were washed in PBS and slides prepared as with S2c. Slides were imaged using a Nikon Dual Cam widefiled microscope with a 60x objective. Excitation at 440 nm and emission was collected in a ruthenium (red/deep red) filter set up on the machine. Images were processed using Image J.

S2h. *Galleria* hemolymph transmission electron microscopy (TEM)

4 larvae were prepared as in S2f for the with AB184 (10^6 CFU/mL) and $\mathbf{1}^{4+}$ 80 mg/kg. Hemolymph extractions were taken at 0, 24, 48, 72 and 96 hours. A pellet was not recovered for the 72 hour time-point. Cells were pelleted (microfuge, 14 000 rpm, 90 s). The cells were fixed using 3 % glutaraldehyde in sodium cacodylate buffer (pH 7.4) and dehydrated using a series of ethanol washes (70 – 100 % ethanol). TEM samples were sectioned in Araldite resin by microtome using a diamond knife. Samples were examined on a FEI Tecnai instrument operating at 80 kV equipped with a Gatan 1 K CCD camera. Images were processed using Image J.

Supplementary 3 – supporting data

S3a. Kaplan Meier survival curves for AB184 and PA2017 10^6-10^8 CFU/mL

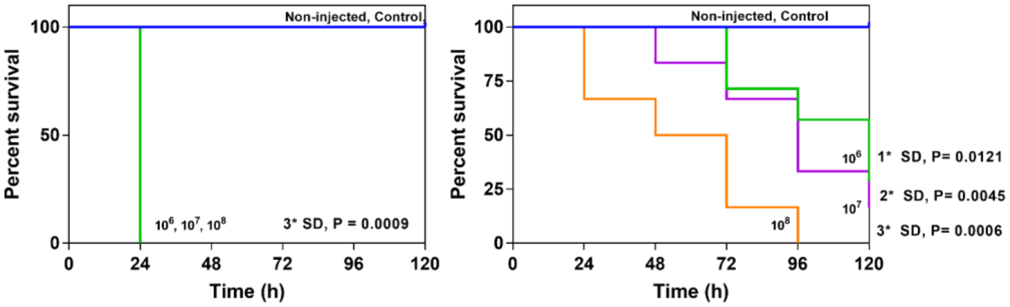

Figure S1| Kaplan Meier percentage survival curves of initial screen, comparing *Galleria* injected with bacteria and controls (no injected, water). Percentage survival for PA2017 (10^6-10^8) (**left**) and AB184 (10^6-10^8) (**right**) are individually compared to the controls for Log-Rank tests and statistical differences and P values are given.

S3b. Images of PA2017 larvae injected with 10^3 CFU/mL

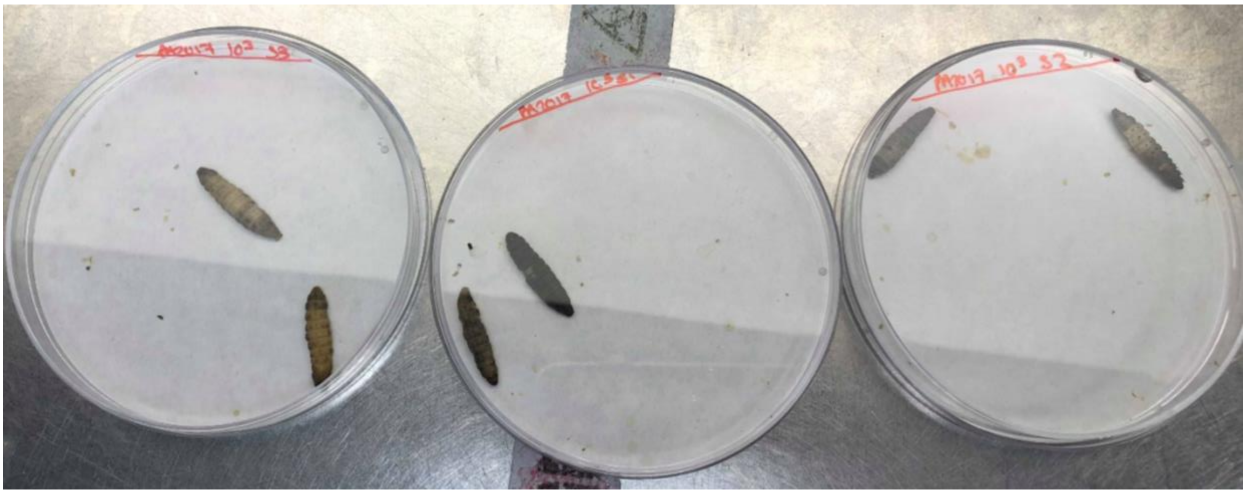

Figure S2| Images of larvae injected with PA2017 (10^3 CFU/mL) at 24 hours. Completely melanised larvae were dead at this time-point.

S3c. PA2017 percentage activity and melanisation

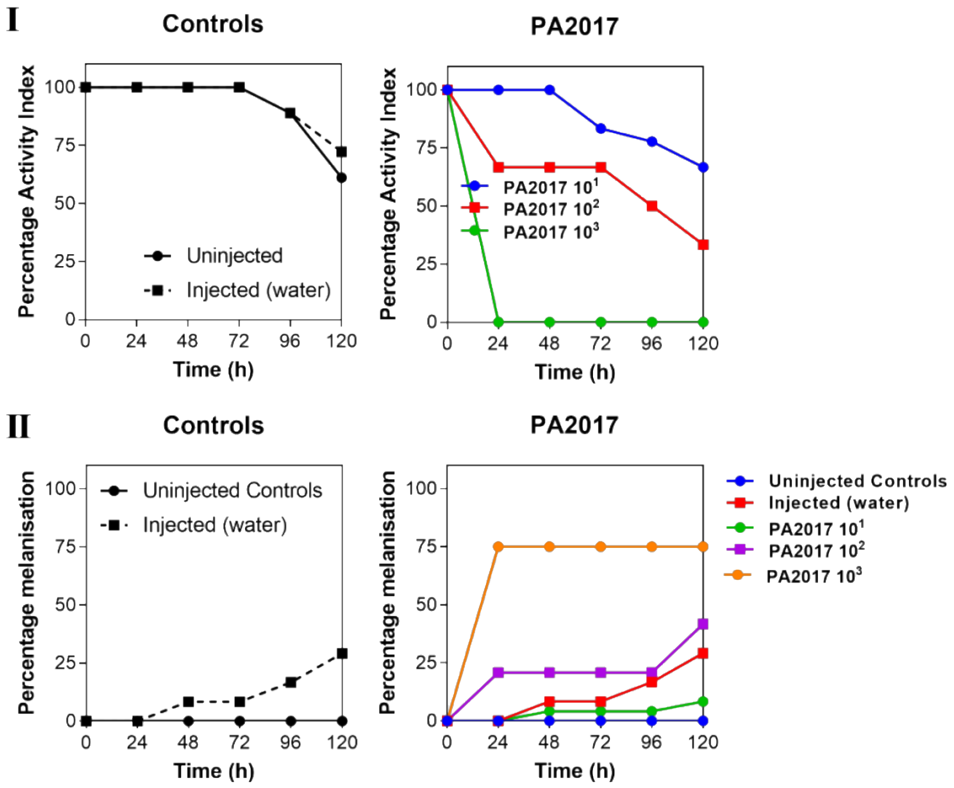

Figure S3| (I) Percentage activity and (II) melanisation scores of *Galleria Mellonella* larvae over 120 hours, scores were taken every 24 hours. Galleria were injected with 10 µL of PA2017 (10^1-10^3 CFU/mL) into their right pro-leg. Larvae were incubated at 37 °C in a petri-dish containing paper.

S3d. Larvae images at 120 hours for larvae infected with AB184 10^2 - 10^5 CFU/mL

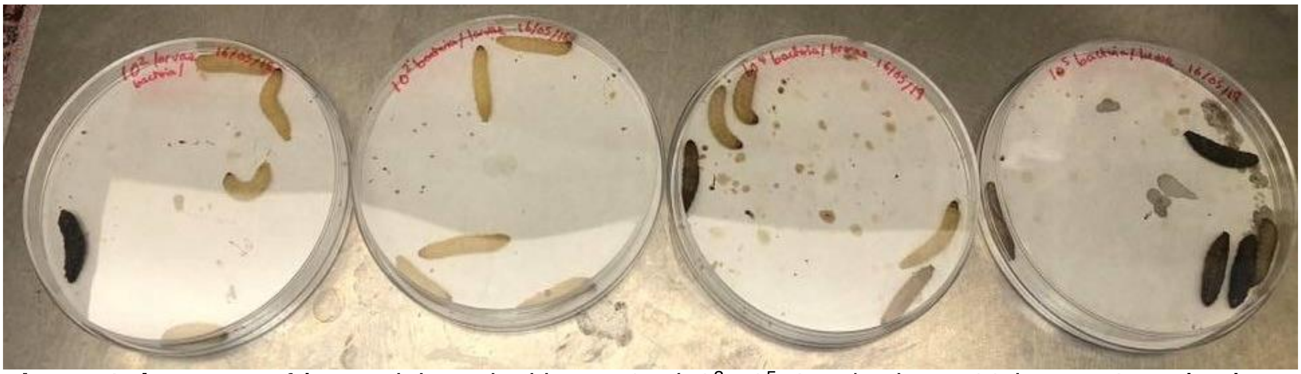

Figure S4| Images of larvae injected with AB184 (10^2-10^5 CFU/mL) at 120 hours. Completely melanised larvae were dead at this time-point.

S3e. AB184 percentage activity and melanisation

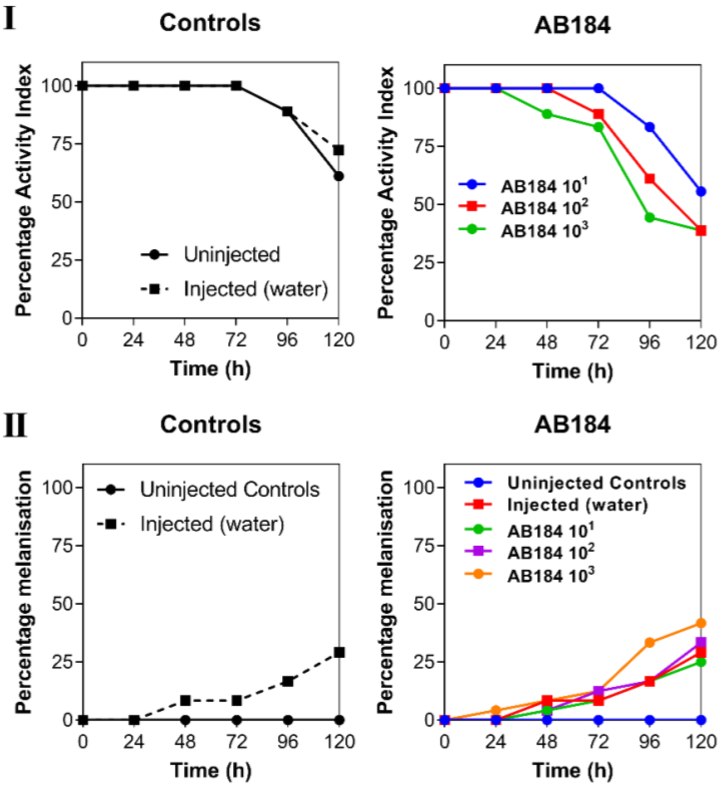

Figure S5| Percentage activity and melanisation scores of *Galleria Mellonella* larvae over 120 hours, scores were taken every 24 hours. Galleria were injected with 10 µL of AB184 (10^1-10^3 CFU/mL) into their right pro-leg. Larvae were incubated at 37 °C in a petri-dish containing paper.

S3f. Compound only controls infection model

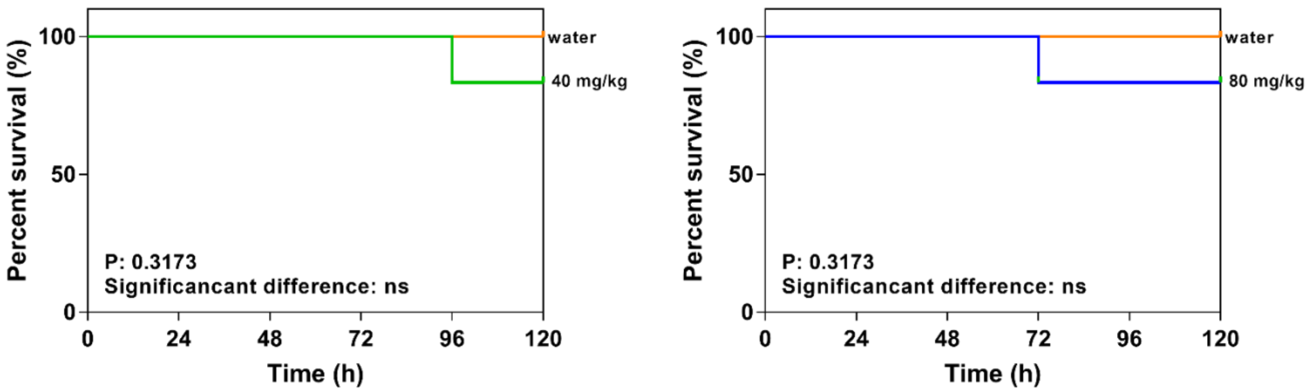

Figure S6| Kaplan Meijer survival curves of *Galleria Mellonella* injected with 10 µL of **1**$^{4+}$ (40, 80 mg/kg) or water incubated at 37 °C in a petri-dish containing paper. Live/dead scores were taken over 24 hours and Log-rank statistical t-tests conducted to determine the significant difference between compound treated larvae and the controls. 7 larvae were injected per condition.

S3g. Larvae images from initial tox screen ½ MIC, MIC and 2MIC concentrations of 1^{4+}

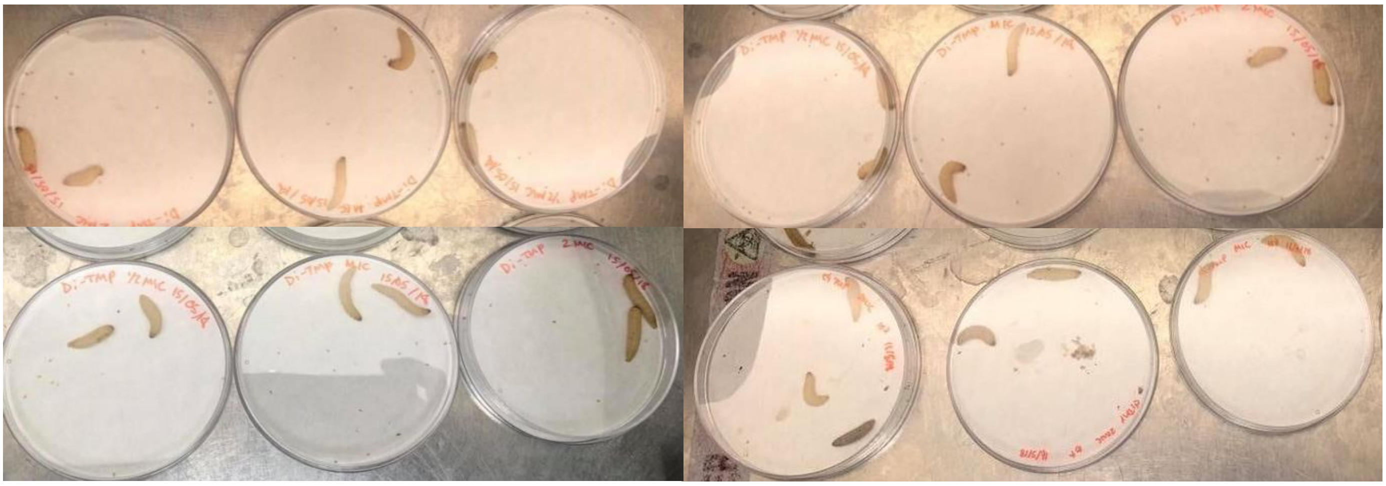

Figure S7| Images of larvae injected with $\mathbf{1}^{4+}$ at ½, 1 and 2 x MIC concentration at 24 (**A**), 48 (**B**), 72 (**C**) and 96 (**D**) hours.

S3h. Activity/melanisation graphs for AB184 10^6 CFU/mL infection model

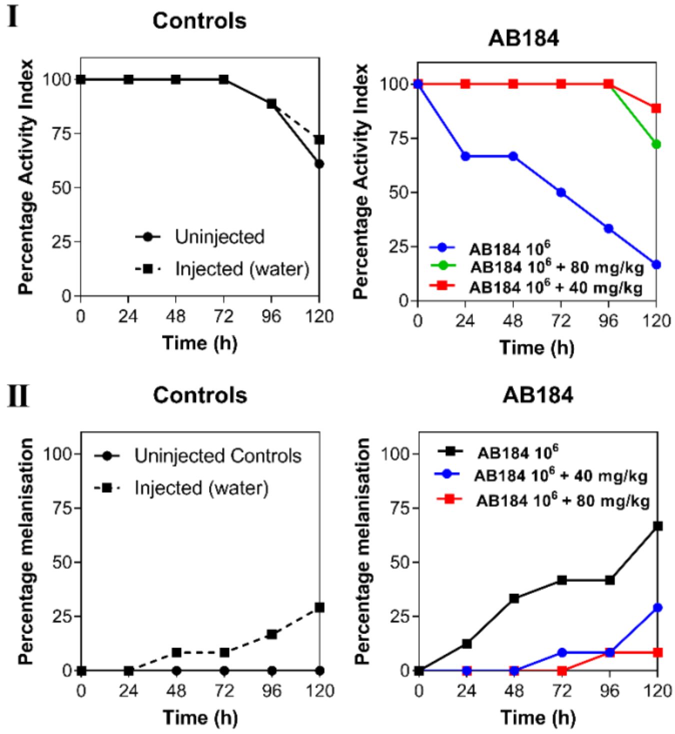

Figure S8| Percentage activity (I) and melanisation scores (II) of *Galleria Mellonella* larvae over 120 hours, scores were taken every 24 hours. Galleria were injected with 10 µL of: AB184 (10^6 CFU/mL), $\mathbf{1}^{4+}$ (40, 80 mg/kg) + AB184 (10^6 CFU/mL), water or non-injected controls. Larvae were incubated at 37 °C in a petri-dish containing paper.

S3i. Kaplan Meier survival curves for AB184 infection model and treatment with 1[4+]

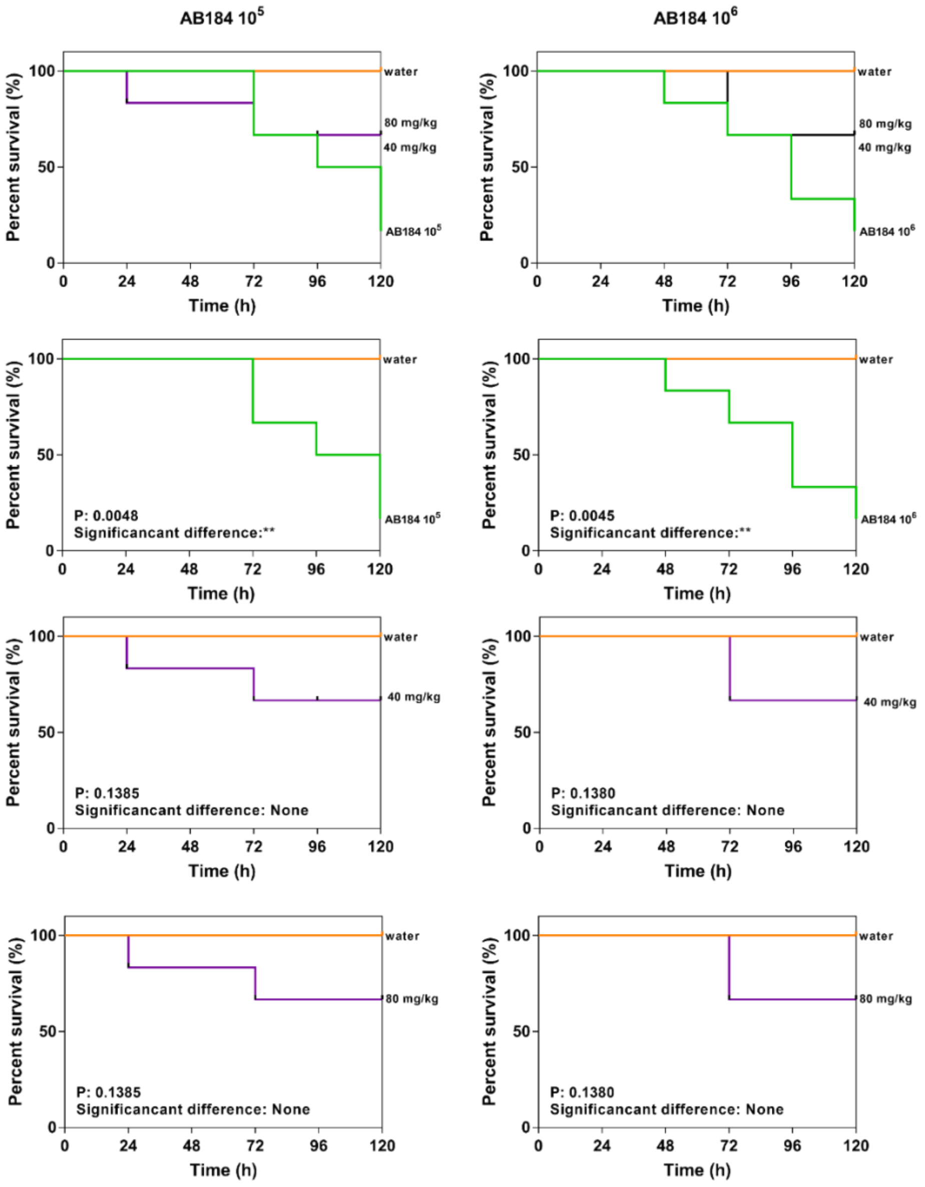

Figure S9| *Galleria Mellonella* infection model. Kaplan Meier percentage survival curves comparing *Galleria* injected with AB184 (green), water (orange) and **1**$^{4+}$ (purple) (**a**). Percentage survival for: AB184 (10^5, 10^6) compared with water (**b**), 40 mg/kg treatment and water (**c**) and 80 mg/kg and water (**d**). Co-injected larvae were injected with bacteria in their right pro-leg then **1**$^{4+}$ 30 minutes later in their left pro-leg. Larvae were incubated for 120 hours at 37.5 °

References

(1) Hornsey, M.; Ellington, M. J.; Doumith, M.; Thomas, C. P.; Gordon, N. C.; Wareham, D. W.; Quinn, J.; Lolans, K.; Livermore, D. M.; Woodford, N. *J Antimicrob Chemother*. **2010**, 65 (8), 1589-1593.

(2) Hall, M. J.; Middleton, R. F.; Westmacott, D.; *J Antimicrob Chemother*. **1983**, 11, 427-433.

(3) Karlowsky, J. A.; Hoban, D. J.; Zhanel, G. G.; Goldstein, B. P. *J Antimicrob Agents*. **2006**, 27 (2), 174-177.

(4) Hornsey, M.; Loman, N.; Wareham, D. W.; Ellington, M. J.; Pallen, M. J.; Turton, J. F.; Underwood, A.; Gaulton, T.; Thomas, C. P.; Doumith, M.; Livermore, D. M.; Woodford, N. *J Antimicrob Chemother*. **2011**, 66 (7), 1499-1500

APPENDIX

^{1}H-NMR SPECTRUM

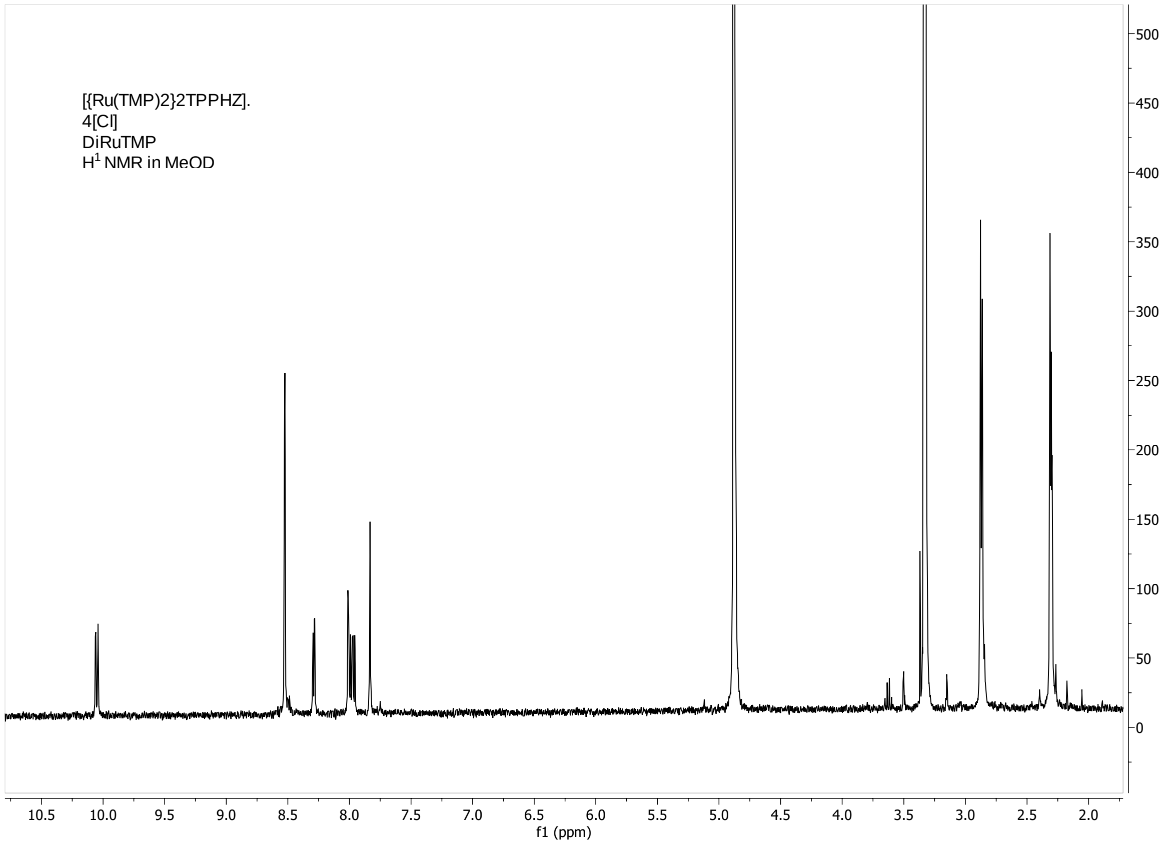

ES-MS

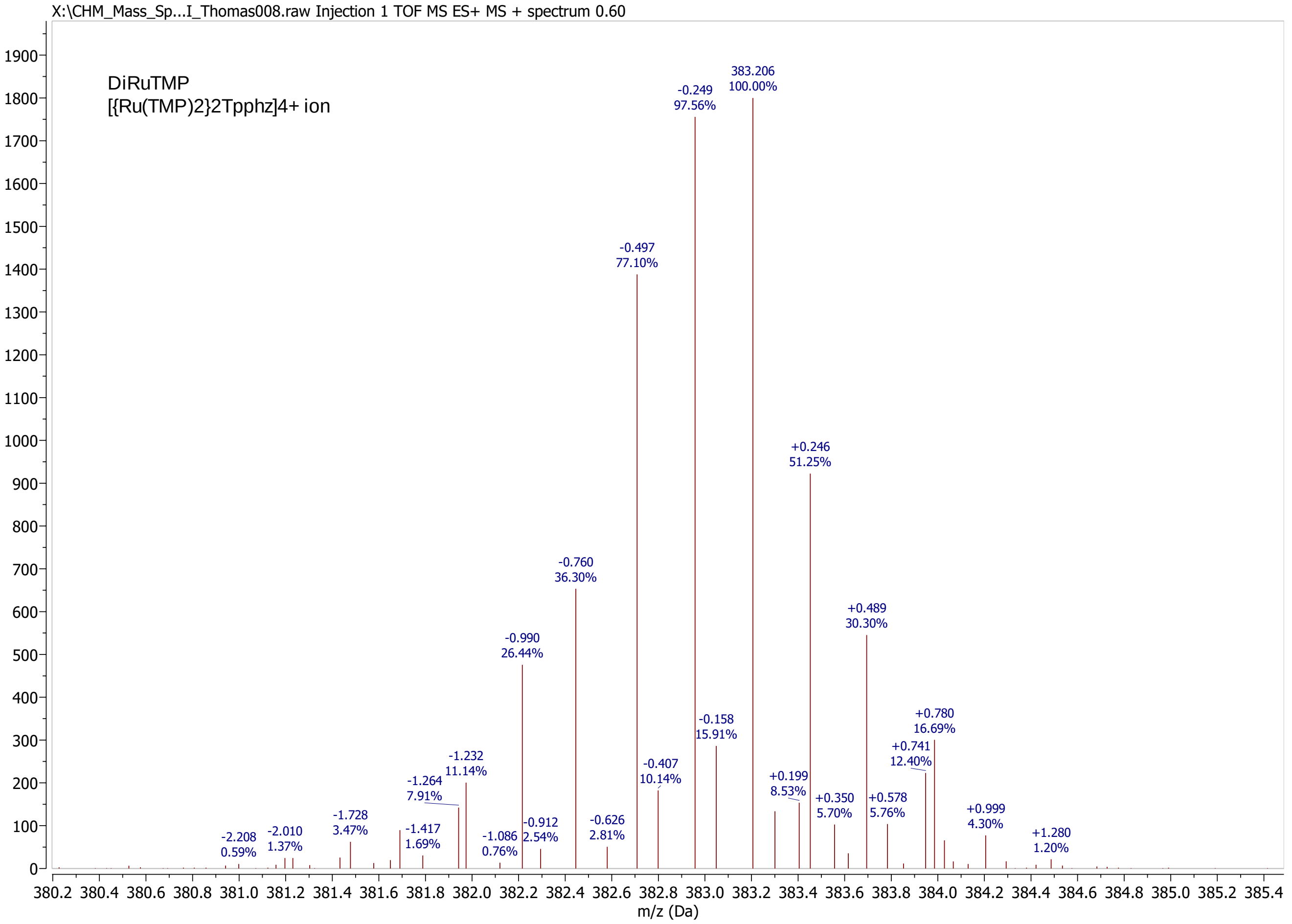
X:\CHM_Mass_Sp...I_Thomas008.raw Injection 1 TOF MS ES+ MS + spectrum 0.60
DiRuTMP
[{Ru(TMP)2}2Tpphz]4+ ion
383.206 100.00%
-0.249 97.56%
-0.497 77.10%
+0.246 51.25%
-0.760 36.30%
+0.489 30.30%
-0.990 26.44%
+0.780 16.69%
-0.158 15.91%
+0.741 12.40%
-1.232 11.14%
-0.407 10.14%
+0.199 8.53%
-1.264 7.91%
+0.578 5.76%
+0.350 5.70%
+0.999 4.30%
-1.728 3.47%
-0.626 2.81%
-0.912 2.54%
-1.417 1.69%
-2.010 1.37%
+1.280 1.20%
-1.086 0.76%
-2.208 0.59%
m/z (Da)
